# Obesity Treatments to Improve Type 1 Diabetes (OTID): a randomized controlled trial of the combination of glucagon-like peptide 1 analogues and sodium-glucose cotransporter 2 inhibitors—protocol for Obesity Treatments to Improve Type 1 Diabetes (the OTID trial)

**DOI:** 10.1186/s13063-024-07930-3

**Published:** 2024-02-16

**Authors:** Ebaa Al-Ozairi, Kavita Narula, Alexander D. Miras, Etab Taghadom, Abeer El Samad, Jumana Al Kandari, Anas Alyosef, Anant Mashankar, Werd Al-Najim, Carel W. le Roux

**Affiliations:** 1 Dasman Diabetes Institute, Kuwait City, Kuwait; 2 Department of Metabolism, Digestion and Reproduction, Imperial College London, London, UK; 3 School of Medicine, Ulster University, Coleraine, UK; 4 Amiri Hospital, Ministry of Health, Kuwait City, Kuwait; 5 Diabetes Complications Research Centre, Conway Institute, University College of Dublin, Dublin, Ireland

**Keywords:** Type 1 diabetes, Obesity, Sodium-glucose cotransporter 2 inhibitor, Glucagon-like peptide 1 receptor analogue, Randomized controlled trial

## Abstract

**Background:**

The guidelines of the American Diabetes Association and European Association for the Study of Diabetes suggest that patients with obesity type 2 diabetics and chronic kidney disease need either glucagon-like peptide 1 receptor analogues or sodium-glucose cotransporter-2 inhibitors. If neither achieve metabolic control, then the recommendation is to combine both drugs. The evidence base for combining glucagon-like peptide 1 receptor analogues and sodium-glucose cotransporter-2 inhibitors is not well researched, and hence, the impact of the guidelines is limited. The aim of this randomized controlled trial is to test the impact of the combination of glucagon-like peptide 1 receptor analogues/sodium-glucose cotransporter-2 inhibitors on body weight and kidney damage, in patients with type 1 diabetes and chronic kidney disease. In addition, we will explore the associated changes in the metabolic pathways with each of the treatments used in this randomized controlled trial.

**Methods:**

In this 6-month randomized control trial, 60 participants aged between 21 and 65 years, with a body mass index above 25 kg/m^2^, and type 1 diabetics with chronic kidney disease will be randomized to receive 1 of 5 possible treatments: (1) standard care (control), (2) glucagon-like peptide 1 receptor analogues alone, (3) sodium-glucose cotransporter-2 inhibitors alone, (4) combination of glucagon-like peptide 1 receptor analogues and sodium-glucose cotransporter-2 inhibitors and (5) combination of glucagonlike peptide 1 receptor analogues and sodium-glucose cotransporter-2 inhibitors with intensive lifestyle advice. The primary objective will be the percentage change in total body weight from baseline at 6 months. The secondary objectives are to compare the change in glycaemia; blood pressure; dyslipidaemia; albuminuria; proportion of participants reaching weight loss of ≥ 5%, ≥ 10% and ≥ 15%; and change in BMI (kg/m^2^) from baseline and change in waist circumference (cm). All the experiments will be conducted at the Dasman Diabetes Institute after approval from the local research and ethics committee.

**Discussion:**

The present randomized controlled trial aims to investigate the impact of the combination of glucagon-like peptide 1 receptor analogues and sodium-glucose cotransporter-2 inhibitors on body weight and kidney damage in patients with type 1 diabetes mellitus and chronic kidney disease, as well as exploring the associated changes in the metabolic pathways with each of the treatments used. This study addresses the current gap in the evidence base regarding the combination of these two drugs, which is particularly relevant given the American Diabetes Association and European Association for the Study of Diabetes guidelines recommending their combined use for patients with obesity, type 2 diabetes, and chronic kidney disease who do not achieve metabolic control with either drug alone.

**Trial registration:**

ClinicalTrials.gov Identifier: NCT05390307 Trial registration date - 25th May 2022

**Supplementary Information:**

The online version contains supplementary material available at 10.1186/s13063-024-07930-3.

## Introduction

### Background and rationale {6a}

As the obesity pandemic continues unabated, one can expect to see an increase in complications in patients with type 1 diabetes (T1D) and type 2 diabetes (T2D), such as chronic kidney disease (CKD) [1]. As a result, early deaths will rise, preceded by an increase in kidney failure, requiring dialysis and renal transplantation [2]. Intensive diabetes therapy aimed at achieving near normoglycemia reduces the risk of complications of T1D [3]. However, glycaemic control remains suboptimal and difficult to achieve with insulin therapy alone, and only a minority of adults with T1D achieve appropriate glycated haemoglobin (HbA1c) goals. Many patients with T1D suffer from hypoglycaemia and weight gain whilst trying to achieve target HbA1c [4].

The guidelines of the American Diabetes Association (ADA) and European Association for the Study of Diabetes (EASD) suggest that patients with obesity, T2D, and CKD need either glucagon-like peptide 1 receptor analogues (GLP1RA) or sodium-glucose cotransporter-2 inhibitors (SGLT2i). If neither achieve metabolic control, then the recommendation is to combine both drugs [5]. The evidence base for combining GLP1RA and SGLT2i is not well developed, and hence, the impact of the guidelines is limited. We will significantly contribute to this proof by distinguishing discrete metabolic pathways or not, triggered by GLP1RA and SGLT2i combinations. Such a discovery has the potential to change clinical practice.

A meta-analysis of 1913 patients in 7 clinical trials with T2D suggests that GLP1RA and SGLT2i combination therapy had a greater reduction in weight of 2.6 kg, HbA1c of 0.6%, and systolic blood pressure of 4.1 mmHg compared to GLP1RA alone and a greater reduction of weight by 1.8 kg, HbA1c by 0.9%, and systolic blood pressure by 2.7 mmHg compared to SGLT2i alone. The studies were not adequately powered to examine CKD or mortality [6].

Additional analysis of Canagliflozin Cardiovascular Assessment Study (CANVAS) in patients with obesity, T2DM and CKD used randomized treatment by subgroup interaction to compare the effects of canagliflozin versus placebo across subgroups defined by baseline use of GLP1RA or not. There were 10,142 patients, of whom 407 (4%) used GLP1RA at baseline. The subgroup of patients with GLP1RA and SGLT2i combinations had the best outcomes as regards to weight loss, glycaemic improvements, and blood pressure changes compared with the other 3 subgroups: (i) no GLP1RA or SGLT2i, (ii) only GLP1RA and (iii) only SGLT2i [7]. This was the first evidence of a potential synergistic effect of combining GLP1RA and SGLT2i, although there are no trial data specifically designed to describe the effects of this combination [8].

The aim of this randomized controlled trial (RCT) is to test the impact of the combination of GLP1RA and SGLT2i on body weight as well as on kidney damage, in patients with T1D and CKD.

### Objectives {7}

The aim of the present study is to compare the clinical effectiveness of patients receiving the following:

- Standard care (control)
- GLP1RA only
- SGLT2i only
- Combination of GLP1RA and SGLT2i
- Combination of GLP1RA, SGLT2i and intensive lifestyle changes

The primary objective will be the percentage change in total body weight from baseline at 6 months. The secondary objectives are to compare the change in waist circumference (cm), glycaemia, blood pressure, dyslipidaemia and albuminuria from baseline, and the proportion of participants reaching weight loss of ≥ 5%, ≥ 10% and ≥ 15% at 6 months.

### Trial design {8}

The OTID study is an open-label, randomized controlled clinical trial. This is a superiority trial in people aged between 21 and 65 years, who have a BMI above 25 kg/m^2^ and have T1D with CKD. The trial will be conducted at Dasman Diabetes Institute in Kuwait. The total duration of participation of follow-up will be 6 months.

## Methods: participants, interventions and outcomes

### Study setting {9}

The study used the SPIRIT reporting guidelines [9]. All the trial activities will be conducted at Dasman Diabetes Institute, a tertiary care for people with type 1 diabetes in Kuwait City, Kuwait, after approval from the local research and ethics committee. All study personnel who have contact with patients must hold the appropriate contracts with the Dasman Diabetes Institute, are appropriately trained on the study protocol and be registered with their appropriate professional bodies in Kuwait. Although obesity also affects young people with type 1 diabetes, our study focused on those aged 21 to 65. Future studies will be able to address those younger and older than our cohort. Potentially eligible patients will be identified by the clinical care team during routine care and invited for screening in outpatient settings. They will be given the patient information leaflet, if the patient expresses an interest in participating, they will be given a minimum of 48 h to consider their participation.

### Eligibility criteria {10}

#### Inclusion criteria

To be considered eligible to participate in this study, a patient must:

- Be aged between 21 and 65 years.
- Have a BMI ≥ 2 5kg/m^2^.
- Have an established diagnosis of T1D (per ADA 2022 definition/criteria) for at least 1 year before the screening visit.
- Insulin treatment for T1D may be either via any Food and Drug Administration-approved continuous subcutaneous insulin infusion pump (CSII) for at least 6 months prior to the screening visit or via multiple daily insulin injections. All participants must be stable on insulin doses/regimens for at least 3 months.
- Have established diagnosis of CKD 1–4.
- Able to give informed consent.

#### Exclusion criteria

Participants will be excluded if:

- They have been treated with GLP1RA or SGLT2i within the last 3 months and/or have a history of GLP1RA or SGLT2i intolerance.
- Diagnosis of T2D or any other type of diabetes (other than type 1).
- Treatment with anti-obesity drugs within 12 weeks prior to randomization.
- Significant changes in the lifestyle (diet or exercise pattern within 3 months of the screening visit).
- Any self-reported changes (gain or loss) in body weight > 5% within 3 months of the screening visit.
- eGFR ≤ 15 mL/min/1.73 m^2^.
- Females of childbearing potential who are pregnant, breastfeeding or intend to become pregnant or are not using or willing to use adequate contraceptive methods during the study period.
- Experienced diabetic ketoacidosis within 6 months of screening visit.
- Experienced severe hypoglycaemia within 6 months of screening visit.
- Any of the following laboratory values at screening (liver chemistry > 3× upper limit of normal, high triglyceride (> 5.7 mmol/L).
- Have a terminal illness or are not primarily responsible for their own care.
- Any other significant disease or disorder which in the opinion of the investigator, may either put the participants at risk or may influence the result of the study or the participant’s ability to participate.
- Untreated or uncontrolled hypothyroidism/hyperthyroidism defined as thyroid-stimulating hormone > 6 mIU/l or < 0.4 mIU/l.
- Family or personal history of multiple endocrine neoplasia type 2 (MEN2) or familial medullary thyroid carcinoma (FMTC).
- Personal history of non-familial medullary thyroid carcinoma.
- History of chronic pancreatitis or idiopathic acute pancreatitis.
- Amylase levels three times higher than the upper normal range.
- Obesity induced by other endocrinologic disorders (e.g. Cushing’s syndrome).
- Current or history of treatment with medications that may cause significant weight gain, within 12 weeks prior to randomization, including systemic corticosteroids (except for a short course of treatment, i.e. 7−10 days), atypical antipsychotics and mood stabilizers (e.g. clozapine, olanzapine, valproic acid and its derivatives, and lithium).
- Initiation of antidepressants during the last 12 weeks.
- Previous surgical treatment for obesity (excluding liposuction if performed > 1 year before trial entry).
- History of other severe psychiatric disorders.
- History of known or suspected abuse of alcohol and/or narcotics.
- History of major depressive episodes during the last 2 years.
- Simultaneous participation in other clinical trials of investigational drugs, lifestyle or physical activity interventions. Patients will only be able to take part following participation in a previous clinical trial after a wash-out period of 16 weeks.
- History of dementia or cognitive impairment.

### Who will take informed consent? {26a}

Based on research governance in Kuwait and our centre, all members of the research team will undergo training on Good Clinical Research Practice (GCP). The screening and consent procedures are performed at the baseline visit. Patients will attend an appointment with a healthcare professional at the study site. The healthcare professional will check their eligibility to participate, explain in detail what the study involves and answer any questions they may have. Full written informed consent to participate will then be obtained. The person obtaining informed consent will be a suitably trained and a competent healthcare professional who, in the opinion of the principal investigator (PI), is able to give a full, unbiased explanation of the study, including benefits and risks, to the potential participant. The person obtaining consent will also have been named in the delegation log of staff as undertaking this duty and will be approved as study personnel by the relevant governance procedures. Each participant will also have a copy of the consent form and patient information leaflet; a copy will be placed in their hospital medical records and the original copy held in the site master file.

### Additional consent provisions for collection and use of participant data and biological specimens {26b}

Not applicable.

### Interventions

#### Explanation for the choice of comparators {6b}

Sixty participants will be randomized to receive one of five possible treatments:

i) Usual standard care
ii) GLP1RA alone
iii) SGLT2i alone
iv) The combination of GLP1RA and SGLT2i
v) The combination of GLP1RA, SGLT2i and intensive lifestyle changes

#### Intervention description {11a}

##### Control (standard)

Participants in the control (standard care) care arm will follow the best standard medical care provided at Dasman Diabetes Institute following the international guidelines for 6 months. Patients with type 1 diabetes receive state-of-the-art care according to the American Diabetes Association and European Association for Study of Diabetes guidelines [10]. Medications will be prescribed by a medical specialist in T1D. In brief, this will involve intensive insulin therapy to maintain good glycaemic control. The treatment is highly individualized, but the three main components of T1D management include insulin therapy, blood glucose monitoring and carbohydrate counting as per the Dose Adjustment For Normal Eating (DAFNE) training programme which patients have received. In addition, patients will also have their hypertension and dyslipidaemia treated whilst being monitored for symptoms and signs of micro- and macrovacular complications of T2D. These participants will not be started on GLP1RA or SGLT2i medication during the trial.

##### GLP1RA alone

Participants in the GLP1RA will be prescribed either liraglutide up to a dose of 3.0 mg daily or semaglutide up to a dose of 1.0 mg subcutaneous injection weekly, depending on their preference. The dose and titration will follow the usual clinical practice. The treatment will last 6 months.

##### SGLT2i alone

Participants in the SGLT2i group will be prescribed dapagliflozin 5–10mg once daily for 6 months.

##### Combination of GLP1RA and SGLT2i

Participants in the combination GLP1RA and SGLT2i group will be prescribed liraglutide up to a dose of 3.0 mg daily or semaglutide up to a dose of 1.0 mg subcutaneous injection weekly plus dapagliflozin 5–10mg for 6 months. The medications will be started simultaneously.

##### Combination of GLP1RA, SGLT2i and intensive lifestyle changes

Participants in the combination GLP1RA and SGLT2i and intensive lifestyle group will be prescribed liraglutide 3 mg once daily or semaglutide 1 mg once weekly subcutaneous injection plus dapagliflozin 5–10mg together with an intensive lifestyle approach for 6 months. Based on age, gender and CKD status, each patient will receive meal plans delivered by a registered dietician. This will involve dietary advice to reduce energy intake with a calorie deficit of 500 calories per day (and may include a period of partial or total meal replacement), accompanied by participation in a physical activity programme, both supported by behavioural change techniques with a regular professional contact. Patients will be cared for in the Dasman Diabetes Institute by a multidisciplinary team consisting of medical specialists in T1D, clinical specialist nurses, dieticians and physical activity specialists. The physical activity programme will include the aim to achieve 150 min of aerobic exercise per week which may include brisk walking or running in those capable of achieving this. The behavioural change approach will be based on cognitive behavioural therapy and will be individualized to each patient as far as possible during one-to-one clinic visits. Group sessions may be considered if it is felt that the patient group recruited would find these valuable. Patients will be seen six times during the study, and at each visit, they will meet the healthcare professional relevant to their treatment allocation for a minimum of 20 min.

#### Criteria for discontinuing or modifying allocated interventions {11b}

Certain circumstances will necessitate stopping the study for a particular participant. Adverse event review and other safety/acceptability assessments will provide the information for the study clinician to withdraw the participant and/or discontinue the treatment drug at any time during the trial.

Participants may withdraw consent from the study before study completion if they decide to do so, at any time and for any reason. If a participant decides to withdraw from the study, this will be recorded in the study records. They will be contacted to thank them for their participation and to inform them that the data collected up to the time point they withdrew will be included in the study analysis and that they will not be contacted again with regard to this study if they so wish. They will not be asked to attend further visits against their will. Furthermore, we will emphasize that their standard care will not be affected by their withdrawal from this study.

Participants will be withdrawn from this study by the research team as agreed by the PI if:

- They are diagnosed with a terminal illness
- The PI, sponsor and or study clinician deem it unsafe for continuation in the study for medical, safety, regulatory or other reasons consistent with applicable laws, regulations or Good Clinical Practice
- They are considered to be lost to follow-up
- Loss of capacity during participation in research
- Treatment with medications that can interfere with the studies

For participants who fail to return to the site, the research team should make a reasonable effort to re-contact the participants (e.g. contacting the participant’s family or family doctor, reviewing available registries or health care databases) and to determine his/her health status, including at least his/her vital status. Attempts to contact such participants must be documented in the participant’s records (e.g. times and dates of attempted telephone contact, receipt for sending a registered letter).

Attempts will be made to assess the primary outcome on all participants whether or not they were compliant and in those who have who have discontinued the treatment. Temporary discontinuation of the treatment drugs may be considered by the PI because of suspected adverse events. A rationale (e.g. severe nausea and vomiting, other gastrointestinal symptoms) must be given in writing by the PI.

Re-initiation of treatment may be done at a lower dose, under close and appropriate clinical/and or laboratory monitoring. For all temporary treatment discontinuations, the duration of treatment discontinuation should be recorded by the research team when considered as confirmed.

Interventions will be discontinued or modified if participants experience any adverse events that are associated with the interventions. The modification will include a reduction in the dose of pharmacotherapy. If the adverse events persist, then pharmacotherapy will be discontinued altogether. Permanent treatment discontinuation is any treatment discontinuation from the PI or the participant, not to re-expose the participant to the allocated drug treatment at any time. Participants may withdraw from treatment with the investigational drug if they decide to do so, at any time and for any reason. The PI may also decide to withdraw a participant from the study based on an inability of the subject to adhere to the obligations of the study or for the safety of the participant. Items that will lead to permanent discontinuation in the study include the following:

- Pregnancy
- Episode of acute pancreatitis and breast malignancy (only for participants in GLP1RA groups)
- They repeatedly violate or are non-compliant with the protocol
- Where GLP1RA or SGLT2i doses are not tolerated by the participant
- Any other contraindication to the study medication which the PI determines to require permanent treatment discontinuation
- All efforts should be made to document the reasons for treatment discontinuation, and this should be documented in the case report form (CRF).

#### Strategies to improve adherence to interventions {11c}

Participants randomized to GLP1RA and/or SGLT2i arm will be instructed to take their subcutaneous injections or tablets whilst continuing their usual medication. Participants will be given specific instructions to adhere to the titration policy of GLP1RA. GLP1RA and/or SGLTi will be prescribed and provided to participants for the duration of the trial.

Participants will be asked to return all unused investigational products and vials/packages to the pharmacy at each study visit. Compliance and concordance with GLP1RA and/or SGLTi will be evaluated and discussed at each study visit based on tolerability and returned pens. Participants will be defined as treatment-compliant if they actually receive at least 70% of planned doses.

#### Relevant concomitant care permitted or prohibited during the trial {11d}

Only the patient in the control group will continue to receive regular standard care during the trial.

Participants in the GLP1RA group will be prescribed either liraglutide up to a dose of 3.0 mg daily or semaglutide up to a dose of 1.0 mg subcutaneous injection weekly, depending on their preference. The dose and titration will follow the usual clinical practice.

Participants in the SGLT2i group will be prescribed dapagliflozin 5–10 mg once daily for 6 months.

Participants in the combination GLP1RA and SGLT2i group will be prescribed liraglutide up to a dose of 3.0 mg daily or semaglutide up to a dose of 1.0 mg subcutaneous injection weekly plus dapagliflozin 5–10 mg for 6 months. The medications will be started simultaneously.

Participants in the combination GLP1RA and SGLT2i and intensive lifestyle group will be prescribed liraglutide 3mg once daily or semaglutide 1 mg once weekly subcutaneous injection plus dapagliflozin 5–10 mg together with an intensive lifestyle approach for 6 months.

#### Provisions for post-trial care {30}

Post-trial care will be standard care as per Dasman Diabetes Institute clinical pathways. As previously described, the Dasman Diabetes Institute clinical pathways include that all people with type 1 diabetes were enrolled in the Dose Adjustment for Normal Eating (DAFNE) programme, a structured education programme for type 1 diabetes. The DAFNE team had regular contact, collected information and recommended therapy changes whilst monitoring adverse events, vitals and laboratory reports. All participants were on insulin treatment using US Food and Drug Administration-approved insulin pumps (continuous subcutaneous insulin infusion) or multiple daily injections. Patients were on stable insulin doses for 3 months. All patients had a blood ketone meter and urine reagent strips, and they were on a flash or continuous glucose monitoring device for at least 2 months [11]. Compensation for harm will be provided by Dasman Diabetes Institute as per research standard operating procedures.

### Outcomes {12}

#### Primary outcome

The primary outcome will be the percentage change in total body weight at 6 months.

#### Exploratory outcomes

Secondary outcomes at 6 months will be the following:

- Proportion of participants reaching total body weight loss of ≥ 5%, ≥ 10% and ≥ 15%
- Change in waist circumference
- Change in HbA1c
- Change in the mean 24-h systolic blood pressure
- Change in the mean 24-h diastolic blood pressure
- Change in lipid profile
- Change in liver biochemistry
- Change in renal biochemistry
- Change in albumin creatinine ratio
- Continuous glucose monitoring parameters

### Participant timeline {13}

#### Invitation of eligible patients

A study clinician will determine the eligibility of potential participants in the clinic and approach suitable participants.

#### Screening and consent visit

The screening and consent procedures are performed at the screening visit. Patients will attend an appointment with a healthcare professional at the study site. All study personnel who have contact with patients must hold the appropriate contracts with the Dasman Diabetes Institute are appropriately trained on the study protocol and registered with their appropriate professional bodies in Kuwait. The healthcare professional will check their eligibility to participate, explain in detail what the study involves and answer any questions they may have. Full written informed consent to participate will then be obtained.

#### Screening visit

A member of the research team will record in the screening visit CRF the participant demographics, medical and surgical history and medication history and perform measurement of anthropometrics (height, weight and waist circumference), blood pressure and pulse rate for all the participants in line with site’s own SOPs. The research team member will arrange for standard laboratory testing (HbA1c, fasting glucose, thyroid stimulating hormone, free T4, lipid profile, renal profile, liver function test and 24-h urine creatinine, microalbumin and sodium) and will also provide participants with urine containers and instructions for the collection of the 24-h urine sample for creatinine clearance test. Any member of the study team who is on the delegation log would be allowed to record data. This is typically a doctor or a clinical nurse specialist.

A member of the research team will schedule all procedures for visit 1 to be performed on the same day (Table 1). This will include the phlebotomy appointment, dual-energy X-ray absorptiometry (DEXA) scan appointment, magnetic resonance imaging (MRI) of the liver appointment, 24-h blood pressure (BP) monitor appointment and the intermittently scanned continuous glucose monitoring (isCGM) appointment.

**Table 1:**
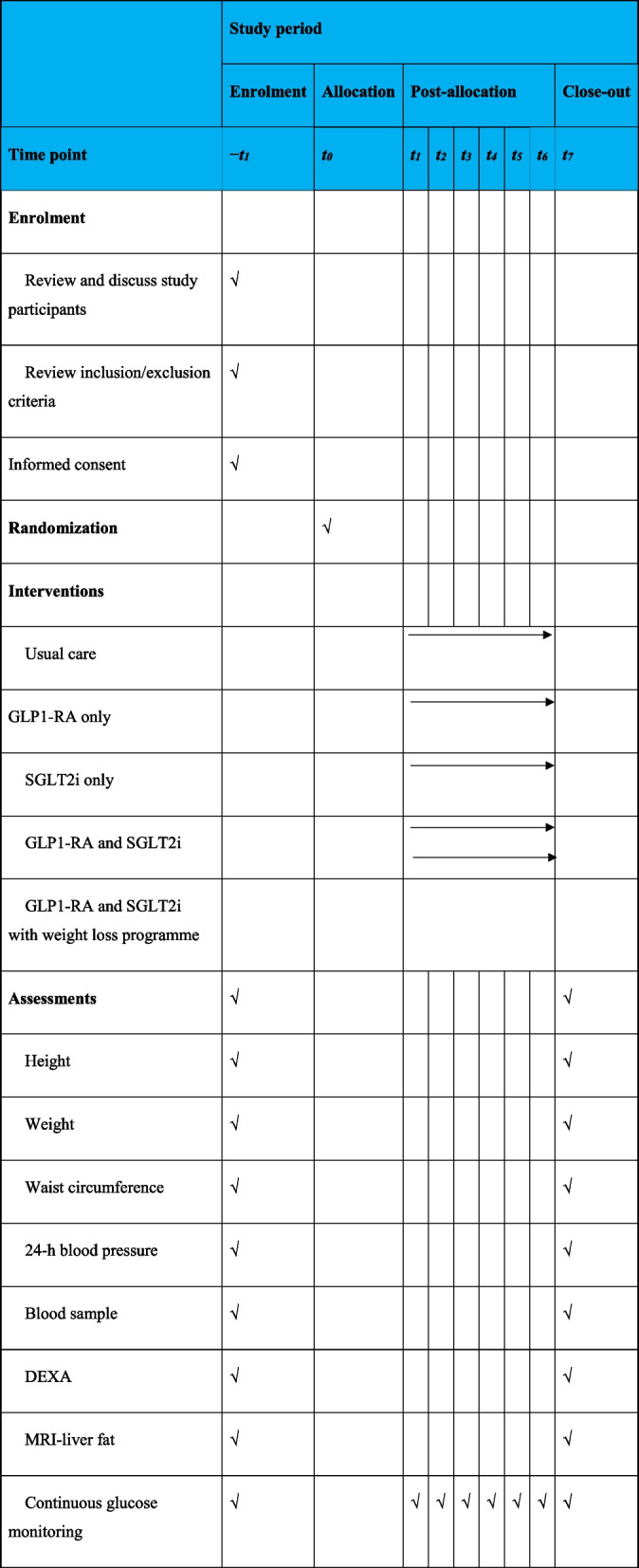
Template for the schedule of enrolment, interventions and assessments for OTID

Before visit 1, all participants will receive two reminder calls to remind them of the date and time of their appointment, as well as instructions for urine collection. Participants must also fast for at least 8 h before the phlebotomy appointment and will be asked to avoid taking calcium, iron and vitamin D supplements for 48 h before the radiological procedure.

##### Visit 1

All participants are provided with blood glucose metres and urine ketone strips to be used for additional monitoring. Instructions will be provided to report any symptoms of hypoglycaemia and measurements of ketones if they were to exhibit any symptoms of diabetic ketoacidosis. Visit 1 includes five procedures:

- Phlebotomy procedure—visit 1

All participants will perform this procedure on the same visit day. Blood for biochemistry and the 24-h urine container will be collected at visit 1 and after 6 months from the intervention at visit 2. HbA1c, fasting glucose, thyroid-stimulating hormone, free T4, lipid profile and liver and renal biochemistry will be measured using standard laboratory methods (Roche Modular system, Roche Ltd.). A urine microalbumin and 24 urine creatinine will also be collected using standard laboratory methods (Enzymatic Roche Cobas 6000 Analyzer).

- DEXA scan—visit 1

All participants will have a DEXA scan performed on the same visit day, to measure fat mass and fat-free mass at visit 1 and after 6 months from the intervention at visit 2. Total fat mass, total lean mass, arm lean mass, leg lean mass and trunk lean mass will be measured using a GE Lunar iDXA scanner.

- Magnetic resonance imaging (MRI) of the liver—visit 1

All participants will have a liver MRI test on the same visit day or within a window of 10 days, to measure fat and fibrosis in the liver at visit 1 and after 6 months from the intervention at visit 2. Liver fat will be quantified by MRI. The proton density fat fraction (PDFF) technique is used to quantify liver fat. The MRI studies are performed on a 1.5-T scanner (Signa Artist, GE Medical Systems, USA). The PDFF calculation is done with the IDEAL-IQ sequence provided by the manufacturer (Slice thickness 8mm, echo time 6 ms, echo repetition time 13.3 ms). This sequence is designed to separate water and triglyceride fat by acquiring six different echoes on the IDEAL (iterative decomposition of water and fat with echo asymmetry and least square estimation) technique with simultaneous T2* correction (Idilman IS, Tuzun A, Savas B, et al.; quantification of liver, pancreas, kidney and vertebral body MRI-PDFF in non-alcoholic fatty liver disease. Abdom Imaging 40:1512-1519 (2015)). The fat fraction of the liver tissue was calculated by placing four rounded regions of interest (ROI) of an average area of 400 mm^2^ in segments II/III, V/VI, VII and VIII of the liver. It was decided to obtain an average fat fraction value of the four readings as the final observation. An additional reference ROI was also placed in the anterior abdominal subcutaneous fat (Idilman IS, Aniktar H, Idilman R, et al.; hepatic steatosis: quantification by proton density fat fraction with MR imaging versus liver biopsy. Radiology 267:767–775 (2013)). Magnetic resonance elastography (MRE) of the liver is performed to assess the liver parenchymal stiffness. This is performed with MR TOUCH sequence (slice thickness 8 mm, echo time 1 ms, echo repetition time 50 ms) provided by the manufacturer (Signa Artist, GE Medical Systems, USA). The Acoustic Driver System (Resoundant Inc. Rochester, MN, USA) is used for impulse generation. In addition, T2-weighted axial sequence was obtained for the upper abdomen to look for any significant incidental findings. Any significant incidental findings observed were conveyed to the PI for necessary remedial action.

- 24-h BP monitoring—visit 1

This procedure is optional and will be conducted at visit 1 and after 6 months from the intervention at visit 2. Twenty-four-hour BP monitorings will be carried out by the staff trained as competent in this procedure [12].

- isCGM procedure—visit 1

All participants will be offered FreeStyle Libre (FSL) 1 sensors for 6 months duration from the start of the intervention till the end of the study. The FSL sensors were installed and connected to the FreeStyle LibreLink App then linked to the cloud-based diabetes management LibreView system.

- Electronic Bluetooth weight scales—visit 1

All participants will be offered eufy Smart Scale C1 and connected to the EufyLife App that automatically syncs all weight results to the app via Bluetooth.

- Randomization—visit 1

Randomization will be carried out by the sponsor at Ulster University (UU) after visit 1 and after confirmation of the participants’ eligibility is received by the research team at Dasman Diabetes Institute. Randomization will be carried out via www.sealedenvelope.com.

#### Follow-up visits

For the majority of participants, follow-up occurs as part of usual care in the Dasman Diabetes Institute, with only two specific study visits added at the start of the study (visit 1) and at 6 months after the study started (visit 2). Adjustments for each participant are made at the discretion and clinical judgement of the investigator, as appropriate for the individuals based on the individuals glycaemic data. All changes in insulin to carbohydrate ratio and/or insulin sensitivity factor will be documented in CRF. For the subgroups randomized to group 02 (GLP1RA alone), group 04 (GLP1RA and SGLT2i combination) and group 05 (GLP1RA, SGLT2i and intensive lifestyle treatment), the empty medication pens, foil packaging and medication compliance will be checked regularly for all participants from the intervention start date until the end of the study.

#### Definition of end of trial

The end of the study is defined as the last participant’s last visit.

### Sample size {14}

Sixty participants will be randomized at a 1:1:1:1:1 ratio to each of the five trial interventions (*n* = 12 per group). Based on the hierarchical analysis model (the “Statistical methods” section), the sample-size calculation assumed at least a 10-percentage-point difference in the mean percentage weight reduction from baseline at 6 months for the combination of GLP1RA, plusSGLT2i plus intensive lifestyle modification as compared with standard care, a common standard deviation of 10% and a dropout rate of 17%. Using these parameters, we will have 90% power to detect statistically significant differences between the two groups at *α* 0.05. This estimation was based on the available evidence for body weight reduction using liraglutide 3mg daily [13] or semaglutide 1 mg weekly [14], dapagliflozin 10 mg daily [15] and the weight loss achieved at the Dasman Diabetes Institute when standard care is provided in people with T1D. The liraglutide trial was a double-blinded multi-centre RCT comparing the effect of liraglutide 3.0 mg vs. placebo in people with obesity. The semaglutide trial was a double-blinded multi-centre RCT comparing the effect of different doses of semaglutide, including the 1 mg equivalent, vs. placebo in people with obesity. We used the best available meta-analysis of trials using dapagliflozin in people with and without type 2 diabetes which specifically focuses on body weight loss. In terms of weight loss, people with type 1 diabetes behave similarly to people with obesity and this is why we used the specific trials/meta-analysis on obesity. The sample size was estimated based only on the primary outcome and not the secondary outcomes which will be exploratory in nature.

### Recruitment {15}

Patients will be identified and approached by their usual clinical care team. They will be given the patient information leaflet; if the patient expresses an interest in participating, they will be given a minimum of 48 h to consider their participation. All the trial activities will be conducted at Dasman Diabetes Institute after approval from the local research and ethics committee.

### Assignment of interventions: allocation

#### Sequence generation {16a}

Randomization will be carried out by the sponsor at Ulster University (UU) on the first visit after screening and after confirmation of the participants’ eligibility is received by the research team at Dasman Diabetes Institute. Randomization will be carried out via www.sealedenvelope.com. Patients will be randomized at a 1:1:1:1:1 ratio to each of the interventions of the trial. Randomization is blocked (using random permuted blocks) to ensure that the groups are balanced periodically.

#### Concealment mechanism {16b}

Randomization will be carried out via www.sealedenvelope.com. The investigator will text the participant’s code to the website and receive the randomization back directly from the website in order to maintain the integrity of the concealment mechanism. The allocation will be announced to the patient at the same visit.

#### Implementation {16c}

Randomization will be carried out via www.sealedenvelope.com, a central randomization system. The investigator will text the participant’s code to the website and receive the randomization back directly from the website in order to maintain the integrity of the concealment mechanism. The allocation will be announced to the patient at the same visit.

### Assignment of interventions: blinding

#### Who will be blinded {17a}

This is an open-label trial. The only staff that will be blinded will be the data analysts.

#### Procedure for unblinding if needed {17b}

Participants and the research team will not be blinded.

### Data collection and management

#### Plans for assessment and collection of outcomes {18a}

The sponsor is responsible for the data management of this study including quality checking of the data. All participant data relating to the study will be recorded on CRFs. The investigator is responsible for verifying that data entries are accurate and correct by physically or electronically signing the CRF. Documentation must be completed in source documents or participant’s medical record. An audit trail will be maintained in the CRF application containing as a minimum: the old and the new data, identification of the person entering the data, date and time of the entry and reason for the correction. The investigator must ensure that data is recorded in the CRF as soon as possible, preferably within 5 working days after the visit. Once data has been entered, it will be available to the sponsor for data verification and validation purposes. If the electronic source data does not have a visible audit trail, the investigator must provide the monitor with signed and dated printouts. In addition, the relevant site staff should be available for discussions at monitoring visits and between monitoring visits (e.g. by telephone). Study monitors will perform ongoing source data verification of critical data points to confirm that data entered into the CRF by authorized site staff are accurate, complete and verifiable from source documents.

During the screening visit, demographic data, medical history and laboratory parameters will be collected by the study team. This will most frequently be done by the clinical nurse specialist but can be done by any member of the team on the delegation log for this task.

#### Plans to promote participant retention and complete follow-up {18b}

Drop-out rates in similar studies run at Dasman Diabetes Institute are approximately 10%. The research staff form excellent relationships with participants who enjoy taking part in research and contributing to the generation of new knowledge. The research staff are enthusiastic and motivate participants. Retaining patients in clinical trials is crucial for the success of the study and the validity of its results. Although we will not be offering Incentives, we will employ the following strategies to help retain our patients:

1. Effective communication to provide clear information about the study’s goals, procedures and potential risks and benefits.
2. The informed consent process will be comprehensive and easy to understand.
3. Patient-centered approach.
4. Regular follow-up reminders.
5. Patient education.
6. Minimize research visit burden.
7. Supportive staff will be empathetic, supportive and responsive to participants’ needs.
8. Patient engagement with regular patient-centered events will be organized to increase engagement and participant retention. This approach creates a “belonging to a group” feeling and has been very effective in previous studies.
9. Peer support.
10. We will also use technology to facilitate communication.
11. Exit strategy, i.e. a plan for participants who wish to withdraw from the trial.

#### Data management {19}

Participants will be assigned a six-digit unique identifier, a subject ID. Any participant records or datasets that are transferred to the sponsor will contain the identifier only. No direct identifiers from the participant are transferred to the sponsor. The participant and any biological material obtained from the participant will be identified by subject ID, visit number and study ID. Source documents provide evidence for the existence of the participant and substantiate the integrity of the data collected. Source documents are filed at the site. Data that are transcribed into the CRF from source documents must be consistent with the source documents, or the discrepancies must be explained.

All research staff will be trained to a GCP standard, and the staff will only complete tasks they are specifically delegated to do. All data will be securely stored according to the guidelines of the Dasman Diabetes Institute. Verification and monitoring of the study and data will be conducted through monitoring procedures of clinical studies. Ultimately, the PI will be responsible for the adequate execution of the study and all the related study issues. The latest version of the Statistical Package for the Social Sciences will be used to analyse the data.

#### Confidentiality {27}

Maintaining patient confidentiality in clinical trials is of paramount importance to protect participants’ privacy and comply with ethical and legal requirements. We will take several measures to ensure patient confidentiality:

1. Informed consent that explicitly outlines the confidentiality protections in place
2. Anonymization
3. Data encryption
4. Access controls
5. Secure storage of samples and records
6. Training,
7. Using unique identifiers for each participant
8. Limit data sharing
9. Secure data transfer
10. Clear data retention policies
11. Audit trails
12. Incident response plan
13. Regulatory compliance
14. Ethics committee oversight

By implementing these measures and maintaining a strong commitment to patient confidentiality, we are confident we can ensure the privacy and trust of their participants whilst meeting ethical and legal obligations.

#### Plans for collection, laboratory evaluation and storage of biological specimens for genetic or molecular analysis in this trial/future use {33}

Samples will be collected through venesection or urine sampling and stored in − 80 freezers at the Dasman Diabetes Institute. No genetic analyses will be performed. All specimens will be collected using the standard operating procedure specified by the clinical laboratory at the Dasman Diabetes Institute.

## Statistical methods

### Statistical methods for primary and secondary outcomes {20a}

Summary statistics for continuous measures will include sample size, mean, SD, median and interquartile range. Summary statistics for categorical measures (including categorized continuous measures) will include sample size, frequency and percentages. Summary statistics for discrete count measures will include sample size, mean, standard deviation, median, minimum and maximum.

Data on treatment adherence, safety (including adverse events) and treatment satisfaction will be summarized and tabulated.

The primary outcome is % total body weight loss from baseline at 6 months and will be compared between the groups based on a hierarchical model.

(i) Difference in % total body weight loss between the combination of GLP1RA plus and SGLT2i plus intensive lifestyle modification group vs. the standard care group. If statistically significant the following comparison will be made.
(ii) Difference in % total body weight loss between the combination of GLP1RA plus and SGLT2i plus intensive lifestyle modification group vs. the SGLT2i alone group. If statistically significant the following comparison will be made.
(iii) Difference in % total body weight loss between the combination of GLP1RA plus and SGLT2i plus intensive lifestyle modification group vs. the GLP1RA alone group. If statistically significant the following comparison will be made.
(iv) Difference in % total body weight loss between the combination of GLP1RA plus and SGLT2i plus intensive lifestyle modification group vs. the combination of GLP1RA and SGLT2i and intensive behavioural modification group.

The primary and secondary outcomes will be analysed upon the ITT and per-protocol population. The ITT population will include all patients randomized to the interventions. The per-protocol population will include all patients who complete the 6-month follow-up without any major protocol deviations.

To ensure the robustness of our results, we will also compare the groups for the primary and secondary outcomes using a mixed model for repeated measures (MMRM), with terms of treatment, visit and treatment-by-visit interaction, and baseline measurement as a covariate. MMRM naturally accounts for missing data assuming that data are missing at random.

Fisher’s exact test will be used to examine the treatment difference in categorical outcomes. The negative binomial regression model will be used for the treatment comparison of discrete count measures. Unless otherwise noted, all tests of treatment effects will be conducted at a 2-sided alpha level of 0.05, and the confidence interval will be calculated at 95%, 2-sided.

### Interim analyses {21b}

No interim analyses are planned.

### Methods for additional analyses (e.g. subgroup analyses) {20b}

No subgroup analyses will be conducted.

### Methods in analysis to handle protocol non-adherence and any statistical methods to handle missing data {20c}

All efforts will be made to prevent the occurrence of missing data. Nevertheless, it is anticipated that withdrawals will occur and hence there will be missing data on primary and secondary efficacy endpoints. We will use mixed-effects modelling, which naturally accounts for missing data assuming that data are missing at random. The number of participants with missing data per variable and reasons will be reported as recommended.

### Plans to give access to the full protocol, participant-level data and statistical code {31c}

Access to the protocol is available through ClinicalTrials.gov. Access to participant-level data and statistical code will be available upon request.

### Oversight and monitoring

#### Composition of the coordinating centre and trial steering committee {5d}

The trial steering committee will be composed of Professor Carel le Roux, Dr. Ebaa Al-Ozairi and Professor Alex Miras. They will meet every 3 months to discuss the progress of the trial and design future strategy.

#### Composition of the data monitoring committee, its role and reporting structure {21a}

A DMC is not needed for this trial as all treatments are routinely available for people with T1D in Kuwait. The aim of this trial is to compare these treatments and their combination. The analyses of the study will take place by independent analysts who will be blinded to treatment allocation.

#### Adverse event reporting and harms {22}

Throughout the OTID study, all the reported adverse events (AE), adverse reactions (AR), severe adverse events (SAE), serious adverse reactions (SAR) and other unintended effects of trial interventions or trial conduct will be collected and assessed. Members of the research team will ask the participants about AEs at each study visit and will complete the participant AE form, which will be a continuous log so as to capture end dates, and the study SAE log.

During and following a patient’s participation in the study, the PI will ensure that adequate medical care is provided to the participant for any AE, including clinically significant laboratory values related to the trial. All serious adverse events and all non-serious adverse events classified as severe or possibly/probably related to the study treatment will be followed up until the participant has recovered, recovered with sequelae or fatal and until all queries have been resolved.

#### Frequency and plans for auditing trial conduct {23}

The sponsor will audit the trial every 6 months according to its standard operating procedures.

#### Plans for communicating important protocol amendments to relevant parties (e.g. trial participants, ethical committees) {25}

All changes to the study protocol will be reviewed by the research ethics committee and then reported to the sponsor and funder. All protocol changes will be communicated to and through ClinicalTrials.gov.

#### Additional consent provisions for collection and use of participant data and biological specimens in ancillary studies, if applicable {26b}

Not applicable.

#### Dissemination plans {31a}

Upon completion of the study, all participants will be informed of the results of the study. The results of the study will be presented in national and international meetings and will be submitted for publication to the relevant peer-reviewed journals. Acknowledgement of any supporting organizations, including funders, will be included.

## Discussion

Not applicable.

## Trial status

Recruitment started on 1 February 2023, and the last patient last visit is expected by 1 December 2023.

### Supplementary Information

**Additional file 1.** 

## Figures and Tables

**Fig. 1:**
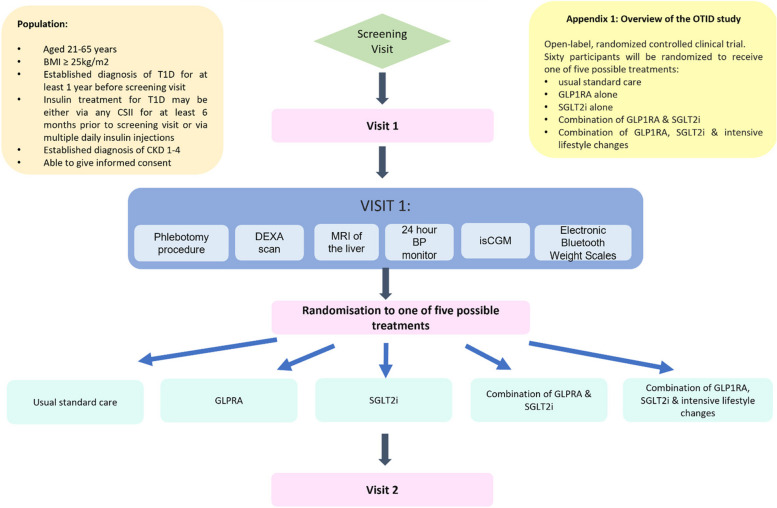
Study flow chart of the OTID trial

## Data Availability

The authors of this manuscript will have access to the final trial dataset. There are no contractual agreements that limit such access for investigators.

## Abbreviations

ADA: American Diabetes Association
AE: Adverse events
AR: Adverse reactions
BP: Blood pressure
CANVAS: Canagliflozin Cardiovascular Assessment Study
CKD: Chronic kidney disease
CRF: Case report form
CSII: Continuous subcutaneous insulin infusion
DAFNE: Dose adjustment for normal eating
DEXA: Dual-energy X-ray absorptiometry
EASD: European Association for the Study of Diabetes
FMTC: Familial medullary thyroid carcinoma
FSL: FreeStyle Libre
GCP: Good Clinical Practice
GLP1RA: Glucagon-like peptide 1 receptor analogues
HbA1c: Glycated haemoglobin
ICH GCP: International Conference on Harmonisation Guidelines for Good Clinical Practice
IDEAL: Iterative Decomposition of water and fat with Echo Asymmetry and Least square estimation
isCGM: Intermittently scanned continuous glucose monitoring
MEN 2: Multiple endocrine neoplasia type 2
MMRM: Mixed model for repeated measures
MOH: Ministry of Health
MRE: Magnetic resonance elastography
MRI: Magnetic resonance imaging
PDFF: Proton density fat fraction
KFAS: Kuwait and Kuwait foundation for advancement of Science
OTID: Obesity Treatments to Improve Type 1 Diabetes
PI: Principal investigator
RCT: Randomized controlled trial
ROI: Regions of interest
SAE: Severe adverse events
SAR: Serious adverse reaction
SGLT2i: Sodium-glucose cotransporter-2 inhibitors
SOPs: Standard operating procedures
T1D: Type 1 diabetes
T2D: Type 2 diabetes
UU: Ulster University
